# An uncommon cause of a common disease: a case report of a rare cause of hypertension

**DOI:** 10.1093/ehjcr/ytae487

**Published:** 2024-09-18

**Authors:** Sarita Rao, Roshan Rao, Achukatla Kumar, Nitika Benjamin, Akshat Pandey

**Affiliations:** Department of Cardiology, Apollo Hospitals Indore, Madhya Pradesh, 452010, India; Department of Cardiology, Apollo Hospitals Indore, Madhya Pradesh, 452010, India; Department of Cardiology, Apollo Hospitals Indore, Madhya Pradesh, 452010, India; Department of Radiology, Apollo Hospitals Indore, Madhya Pradesh, 452010, India; Department of Rheumatology, Apollo Hospitals Indore, Madhya Pradesh, 452010, India

**Keywords:** Secondary hypertension, Renal artery stenosis, Aneurysm, Takayasu arteritis, Case report

## Abstract

**Background:**

Severe hypertension in young patients presents a significant diagnostic dilemma, and treatment can often be codified. Therefore, it is crucial to diagnose these cases for probable secondary hypertension. Common causes of secondary hypertension include large vessel vasculitis, renal artery stenosis, coarctation of the aorta, and endocrine disorders.

**Case summary:**

A 23-year-old Asian male, who was previously in good health, presented with symptoms of chest pain, shortness of breath on exertion grade II, and generalized weakness. On examination, his blood pressure was markedly elevated at 200/110 mmHg. Diagnostic investigations revealed significant vascular involvement, including bilateral renal artery stenosis accompanied by aneurysm formation, celiac trunk disease, and osteal stenosis of the superior mesenteric artery. The patient underwent successful interventional procedure, including renal angioplasty, stenting, and aneurysm coiling. This was followed by tailoring of medical management along with anti-inflammatory and disease-modifying drugs.

**Discussion:**

The diagnosis of Takayasu arteritis (TAK) in this case is supported by the patients’ age, presentation, and imaging according to the new TAK classification criteria by the American College of Rheumatology/European League Against Rheumatism (EULAR) and emphasizes the potential benefits of a pharmaco-invasive approach for optimal outcomes.

**Learning points**

- Bilateral renal artery stenosis with aneurysm formation is rare. Neurofibromatosis and vasculitis should be suspected in such patients.
- In young hypertensive patients, we should look for Type IV Takayasu arteritis, which is a rare and treatable cause of hypertension and can be easily treated with stenting and disease-modifying drugs. Focused treatment of any treatable cause of hypertension at this age usually cures the hypertension.

## Introduction

Hypertension is the leading contributor to cardiovascular morbidity and mortality worldwide.^1^ Arterial hypertension is a significant risk factor, and its prevalence in the general population ranges from 10% to 40%, depending on age and population-specific factors.^2^ Secondary hypertension, which has an identifiable underlying cause, is less common than primary hypertension but tends to be overlooked. It is more prevalent among younger patients and those with resistant or refractory hypertension. In various populations, up to 5% of cases can be attributed to secondary hypertension due to large vessel vasculitis, renal artery stenosis, coarctation of the aorta, primary aldosteronism, pheochromocytoma, and chronic kidney disease.^3,4^ This report describes an uncommon case of secondary hypertension in a 23-year-old male that was characterized by multiple osteal stenoses of the abdominal aortic branches and bilateral renal artery aneurysms.

## Summary figure

**Figure:**
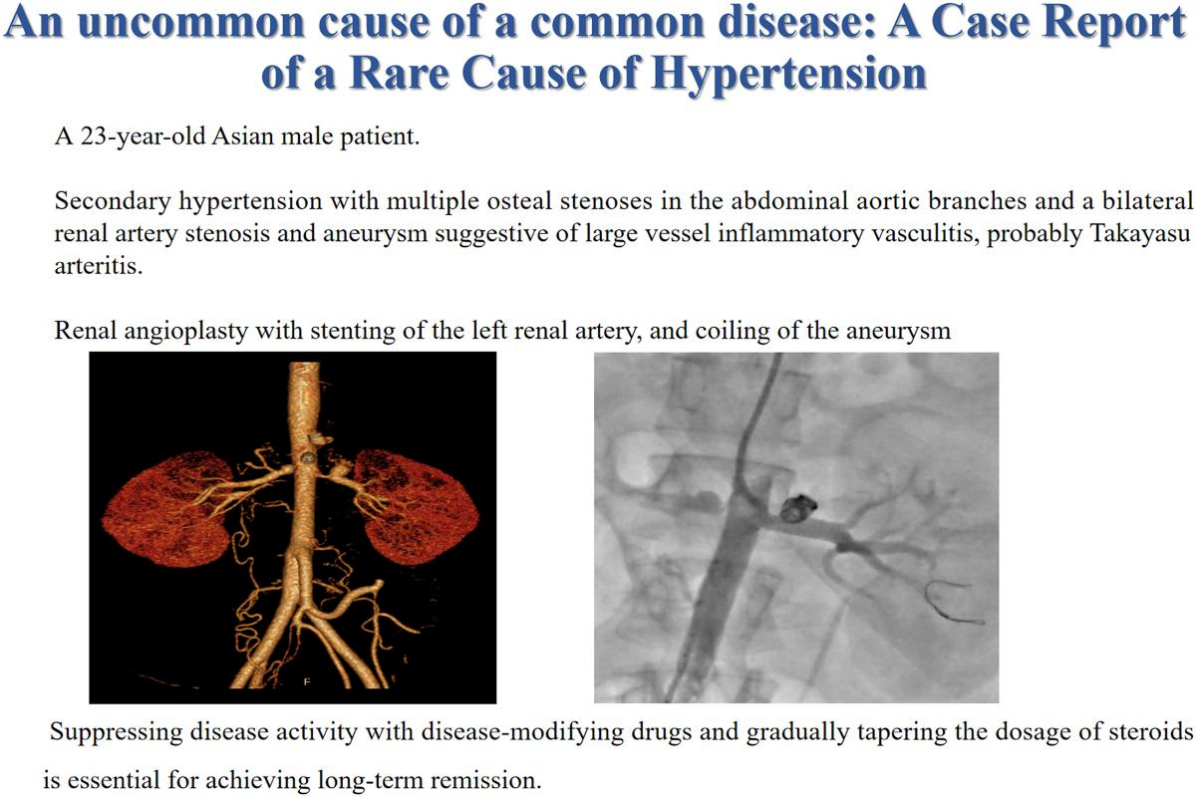


## Case presentation

A 23-year-old previously healthy Asian male presented with a 2-day history of chest pain and weakness. On initial evaluation, he was found to have a blood pressure of 240/120 mmHg and a heart rate of 110/min. His respiratory rate was 20/min, and his temperature was 97°F. Physical examination revealed a soft abdomen, clear bilateral chest, normal heart sounds, and no abnormal neurological findings, and there was no skin manifestation of systemic diseases. His body mass index was 27.34 kg/m². The patient had no significant past medical history. Initial laboratory investigations revealed a haemoglobin level of 15.5 g/dL, a platelet count of 348 000/μL, and a white blood cell count of 10 300/μL. His glucose level was 106.3 mg/dL, creatinine level was 0.84 mg/dL, sodium level was 139 mmol/L, and his potassium level was 4.1 mmol/L. His lipid profile included triglycerides at 95 mg/dL, LDL cholesterol at 100.3 mg/dL, and HDL cholesterol at 40 mg/dL. Cardiac evaluation revealed a negative troponin I test.

**Figure 1:**
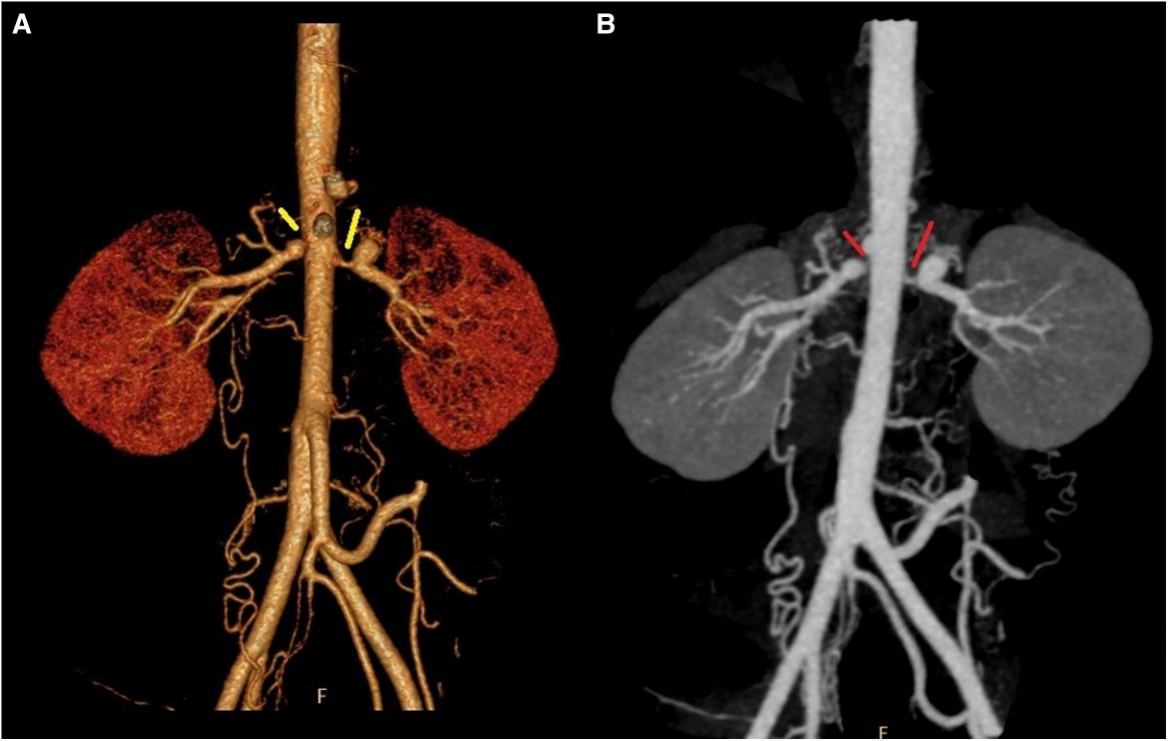
Computed tomography angiography. *A*, *B*) Coronal plane showing the right and left renal artery aneurysm with tight osteal stenosis. Computed tomography Abdomen reveals severe osteoproximal left renal artery stenosis with aneurysm formation. Moderate to severe stenosis of right ostioproximal stenosis with small aneurysm formation.

**Figure 2:**
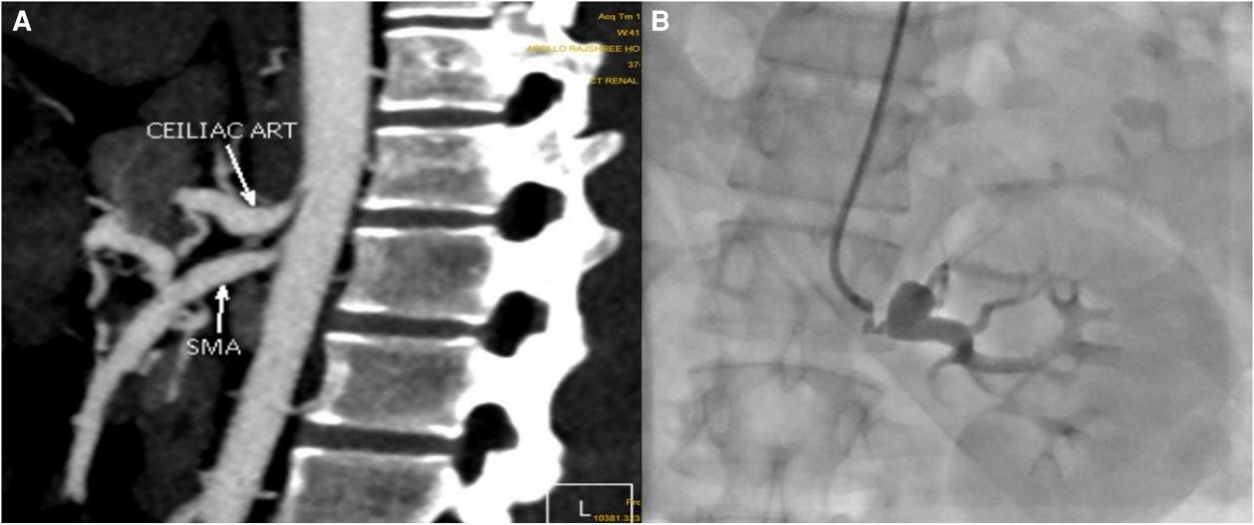
Osteal stenosis of celiac, mesenteric, and renal arteries. *A*) Sagittal view showing the osteal stenosis of celiac and superior mesenteric artery. *B*) Peripheral angiography showing an osteal stenosis of left renal artery with large aneurysm, multiple osteal stenosis with aneurysm points at Takayasu arteritis Type IV.

An electrocardiogram revealed sinus tachycardia and left ventricular hypertrophy. Echocardiography revealed concentric left ventricular hypertrophy and normal left ventricular systolic function with an ejection fraction of 65%. Abdominal ultrasound revealed no significant abnormalities. Computed tomography (CT) angiography of the thoracic aorta revealed a normal thoracic aorta, arch, and neck arteries. Doppler ultrasound of the renal arteries revealed bilateral tardus parvus spectral waveforms, with ∼90% stenosis at the origin of the left renal artery (*Figure 1*). Computed tomography abdominal angiography confirmed high-grade osteal stenosis (80–90%) of the celiac trunk, superior mesenteric artery, and both renal arteries, with an associated post-stenotic saccular aneurysm in the left renal artery measuring 9.0 × 8.0 mm in size. Extensive collateral formation was observed in the retroperitoneal region, gastro-oesophageal junction, and abdominal cavity, predominantly originating from an enlarged inferior mesenteric artery. Peripheral angiography further revealed bilateral renal artery stenosis of 90% with aneurysms and 90% stenosis of osteoproximal celiac trunk (*Figure 2*). Blood analysis revealed normal levels of angiotensin-converting enzyme (31.90 U/L), an antinuclear antibody-negative status, a double-stranded DNA-negative status, a C-reactive protein concentration of 9.37 mg/L, and an erythrocyte sedimentation rate (ESR) of 21 mm/1 h, and all immunological levels were normal.

**Figure 3:**
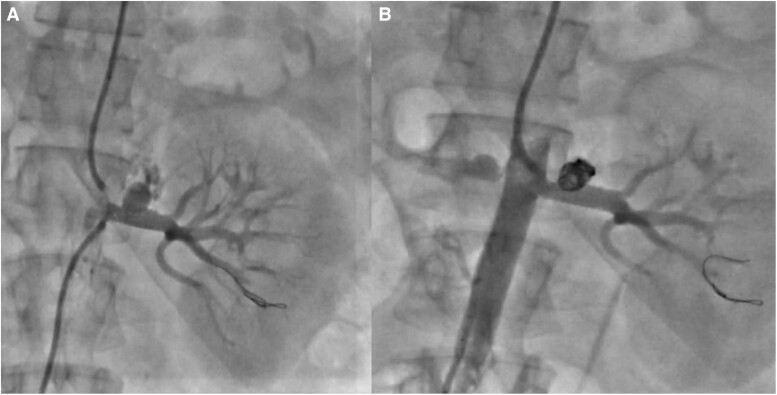
Peripheral angioplasty of left renal artery stenting followed by coiling. *A*) Showing post-stenting of left renal artery did not result in collapse of aneurysm. *B*) Showing post-stenting, the aneurysm was occluded with coil, and there was total occlusion of aneurysm.

The diagnosis of Takayasu arteritis (TAK) in this patient was supported by the patient's presentation, which aligns with Type IV involvement according to the new TAK classification criteria of the American College of Rheumatology (ACR)/European League Against Rheumatism (EULAR). On the second day after admission, the patient underwent successful renal angioplasty with stenting of the left renal artery and coiling of the aneurysm using a 7.0 × 18 mm renal stent and 10 × 30 mm coil (*Figure 3*). The procedure was uncomplicated, after which the patient was transferred to the intensive care unit for observation. After intervention, the patient's vital signs stabilized, and his clinical status improved. A multidisciplinary approach involving intensive care, atorvastatin, clopidogrel, aspirin, prazosin, bisoprolol, amlodipine, and cefotaxim and supportive measures were implemented. The patient was discharged on the fifth day in stable condition. At discharge, his vital signs included a normal temperature, a pulse rate of 84/min, and a blood pressure of 110/70 mmHg. For continued management, he was prescribed a medication regimen including aspirin 75 mg OD, clopidogrel 75 mg OD, atorvastatin 20 mg OD, prazosin 5 mg OD, amlodipine 5 mg OD, bisoprolol 5 mg OD, methotrexate 7.5 mg OD per week, and supportive care.

## Discussion

Takayasu arteritis derives its name from the Japanese professor of Ophthalmology, Mikito Takayasu.^5^ This condition predominantly affects women, accounting for nearly 90% of cases, and typically manifests before the age of 40.^6^ Takayasu arteritis can initially present as uncontrolled hypertension, mainly when the renal arteries are affected, even in the absence of other apparent symptoms. Studies indicate that hypertension occurs in 33–83% of TAK patients worldwide, particularly those with an earlier disease onset.^7–9^ A study by Watanabe *et al*.^10^ indicated that the prevalence rate of Type IV is 5.9% and it is the lowest compared with other types of TAK.

**Figure 4:**
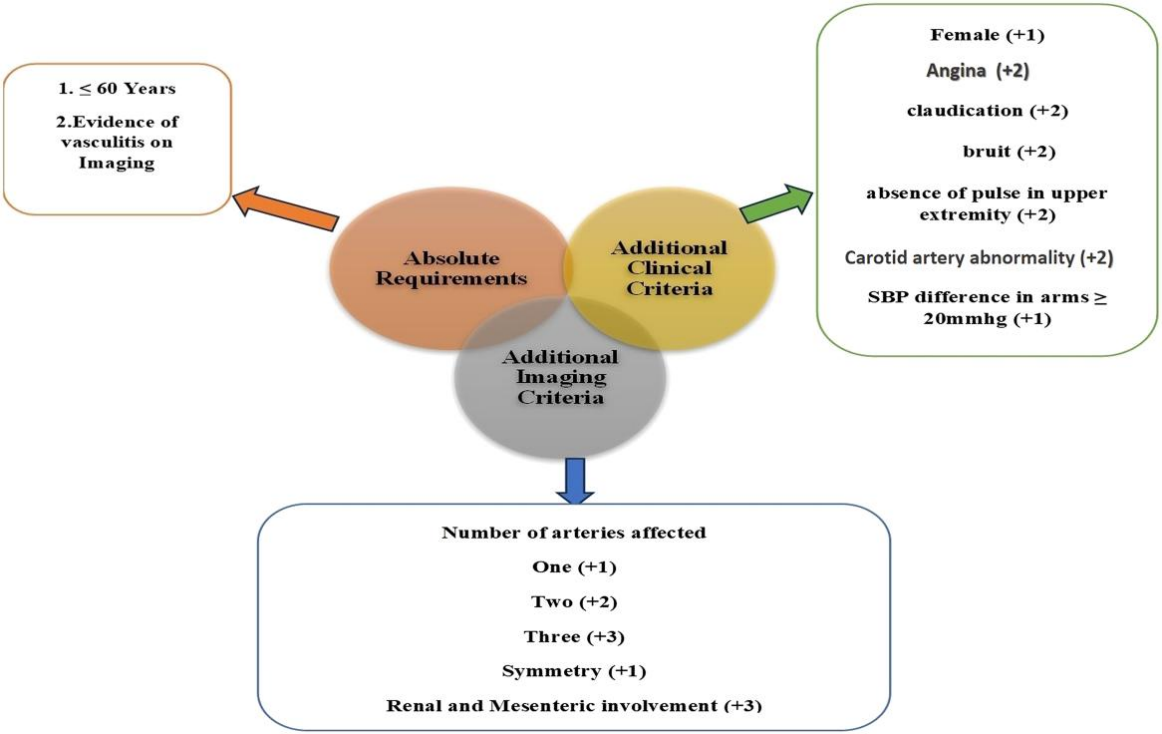
New Takayasu arteritis classification criteria by the American College of Rheumatology/European League Against Rheumatism (EULAR) (2022). *A classification of Takayasu arteritis requires a total score of ≥5 points.

Diagnosing TAK is challenging since no specific laboratory markers or biopsy findings exist. The absence of distinctive constitutional symptoms often leads to delays in diagnosis, with an average duration of ∼1.3 years from symptom onset.^11^ Vascular imaging plays a crucial role in childhood-onset TAK. Angiography is regarded as the gold standard for evaluating vascular lesions associated with TAK. The new 2022 ACR/EULAR classification criteria for TAK include imaging characteristics as an absolute requirement (*Figure 4*).

The diagnosis of TAK was based on the new ACR/EULAR criteria, considering the patient's age, multiple abdominal aortic branch involvement, and accelerated hypertension. The patient experienced chest pain linked to ischaemia, meeting additional clinical criteria for TAK. Imaging showed involvement of the abdominal aorta, renal, and mesenteric arteries, with no significant stenosis in the carotid and subclavian arteries, excluding atherosclerosis and fibromuscular dysplasia. The angiographic classification indicated a rare Type IV involvement (abdominal aorta and renal arteries). (Supplementary material online, *Figure S1*).^12^

Conventional angiography, CT angiography, and renal artery Doppler were used in this patient. The intricate vascular manifestations comprised 90% bilateral renal artery stenosis associated with an aneurysm, 90% osteoproximal celiac trunk disease, and 90% stenosis of the superior mesenteric artery. In light of the normal ESR, vascular intervention was pursued to control symptoms and blood pressure. Both renal arteries were stented at different intervals (right renal was done after 3 months of left renal angioplasty).

Treatment involves a combination of medication and, if necessary, surgical intervention. Glucocorticoids are the primary therapy, providing relief in 60–80% of patients, although recurrence is common during dose reduction in half of patients.^13^ Transcatheter vascular interventions, such as percutaneous transluminal angioplasty and stenting, are critical for managing arterial stenosis in critical areas or if ischaemic symptoms develop, aiming to restore blood flow.^14^

## Conclusion

This case emphasizes the need to consider rare causes, like TAK, in common conditions such as hypertension, highlighting the importance of a thorough diagnostic approach. The management of childhood TAK-induced accelerated hypertension followed the latest ACR/EULAR criteria, showcasing the necessity of a multidisciplinary approach for diagnosing and treating this rare vasculitis.

## Supplementary Material

ytae487_Supplementary_Data

## Data Availability

The data underlying this article will be shared on reasonable request to the corresponding author.

## Lead author biography

Dr Sarita Rao, a trailblazer in interventional cardiology, serves as the Senior Interventional Cardiologist and Director of Cathlab at Apollo Hospitals, Indore, President WINCARS Association, Co-Chair EAPCI International Affairs. With over 23 years of dedicated service, she has achieved numerous milestones, including being the first woman in India to do Renal denervtion therapy, implant a Bioresorbable Vascular Scaffold and perform a Live TAVI case in SAARC countries. Dr Rao's pioneering work extends to the first successful impella surgery and IVL done by a women in India, and the first hybrid procedure for Aortic Aneurysm in Central India. She received numerous awards and published various manuscripts in international journals. Her leadership and expertise have significantly advanced cardiac interventions nationally and internationally.

## Notes

**Consent:** A written informed consent document was obtained from the patient for publication and compliance with the COPE guidelines.

**Funding:** None declared.
