# Supplementary material for: An uncommon cause of a common disease: a case report of a rare cause of hypertension
Source: Eur Heart J Case Rep. 2024 Sep 18;8(10):ytae487. doi: 10.1093/ehjcr/ytae487 (PMC11450471; doi:10.1093/ehjcr/ytae487)
Supplement: ytae487_Supplementary_Data [file ytae487_supplementary_data.docx]

Supplementary Material:

**Figure: Angiographic classification of TAK**
